# From Norovirus to Andes Hantavirus: Indirect Viral Transmission and Emerging Prevention Strategies for Cruise Ship and Confined Environments

**DOI:** 10.1002/rmv.70180

**Published:** 2026-06-30

**Authors:** Umme Laila Urmi, Mark D. P. Willcox

**Affiliations:** ^1^ School of Optometry and Vision Science University of New South Wales Sydney New South Wales Australia

**Keywords:** Andes hantavirus, indirect transmission, norovirus, passive infection control

## Abstract

Cruise ships and other confined travel settings remain highly vulnerable to infectious disease outbreaks. During 2025–2026, multiple norovirus outbreaks and the rare Andes hantavirus outbreak associated with the MV Hondius cruise highlighted the continuing public health challenges posed by both highly transmissible enteric viruses and emerging zoonotic pathogens. This review summarises recent cruise‐associated viral outbreaks with particular emphasis on indirect transmission pathways, environmental persistence, and the limitations of conventional outbreak‐control strategies. Norovirus outbreaks continued to occur despite implementation of enhanced sanitation and infection‐control measures, highlighting the difficulty of interrupting transmission in environments where contaminated surfaces, aerosolised particles, and shared spaces may contribute to viral spread. In contrast, the Andes virus outbreak emphasised the additional risks associated with expedition‐style travel, delayed symptom onset, zoonotic exposure, and complex international surveillance requirements. Current outbreak responses remain largely dependent on reactive measures such as cleaning, disinfection, passenger isolation, and contact tracing. However, the repeated occurrence of these outbreaks suggests that these approaches alone may be insufficient for long‐term prevention in highly interconnected environments. Emerging environmental intervention strategies, including advanced air decontamination systems, antiviral surface coatings, self‐disinfecting smart materials, and peptide‐based antiviral technologies, have demonstrated promising antiviral activity against a range of respiratory and enteric viruses. These technologies may offer complementary passive protection approaches capable of reducing environmental contamination and minimising indirect viral transmission. Collectively, this review highlights the need to move beyond conventional reactive sanitation measures towards integrated and multidisciplinary preparedness strategies for future outbreak prevention in cruise ships and other crowded, closed, and interconnected settings.

## 1 Introduction

Cruise ships and other confined travel modes represent unique ecosystems for the rapid transmission of infectious diseases due to crowded populations, prolonged close interpersonal interactions, shared dining and recreational facilities, and extensive international mobility [1]. Over the past decades, cruise‐associated outbreaks have repeatedly demonstrated how confined and highly interconnected settings can facilitate rapid dissemination of viral pathogens among passengers and crew members, often leading to substantial public health and economic consequences [1, 2]. Among these, norovirus has remained the most frequently reported viral pathogen linked to cruise ship outbreaks worldwide because of its extremely low infectious dose, high shedding rate, environmental persistence, and ability to spread through multiple transmission pathways, including contaminated surfaces, food, aerosols, and person‐to‐person contact [3, 4, 5]. Despite implementation of rigorous sanitation measures and infection‐control protocols, recurrent norovirus outbreaks continue to occur across cruise liners, highlighting the persistent challenges associated with controlling indirect viral transmission in confined environments.

In addition to highly transmissible enteric viruses, the emergence and re‐emergence of zoonotic pathogens have further increased concerns regarding infectious disease preparedness in global travel settings. The recent Andes hantavirus outbreak associated with the MV Hondius cruise in 2026 highlighted how expedition‐style travel and ecotourism activities may introduce emerging pathogens into confined environments, complicating outbreak detection, surveillance, and containment efforts [6]. Although Andes virus transmission differs substantially from norovirus, the outbreak emphasised the vulnerability of cruise‐associated settings to complex transmission dynamics involving delayed symptom onset, environmental exposure, international passenger dispersal, and the potential for limited human‐to‐human transmission. Similar concerns were evident during COVID‐19 outbreaks on cruise ships, where confined environments facilitated rapid viral spread and complicated outbreak containment efforts [7, 8].

Collectively, these outbreaks demonstrate that closed travel environments are not only susceptible to recurrent gastrointestinal outbreaks but may also serve as amplification settings for emerging infectious threats with potentially severe public health implications. Increasing evidence indicates that environmental persistence of viral particles on high‐touch surfaces, aerosolised dissemination within enclosed spaces, and contaminated shared environments play major roles in sustaining transmission, particularly for viruses capable of prolonged environmental survival [9, 10]. These challenges highlight the growing need for innovative and proactive environmental intervention strategies capable of providing continuous passive protection against viral spread. Recent advances in environmental decontamination technologies and antiviral surface engineering have generated considerable interest as potential complementary approaches for reducing viral persistence and minimising indirect transmission risk. This review summarises recent cruise ship‐associated outbreaks involving norovirus and Andes hantavirus during 2025–2026 and highlights the emerging environmental decontamination technologies and antiviral surface modification approaches that may contribute to future outbreak prevention and preparedness in cruise ships and other crowded, closed, and highly interconnected environments.

## 2 Cruise Ship Related Viral Outbreaks During 2025–2026

**FIGURE 1:**
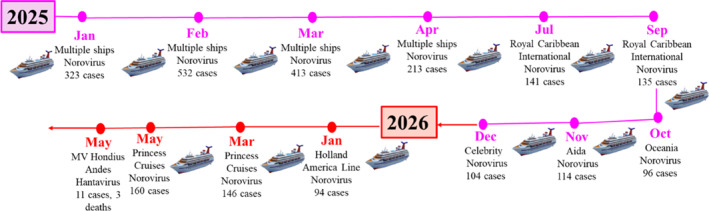
Reported cruise ship viral outbreaks across 2025–2026 [11, 12].

Table 1 summarises some of the most recent travel‐associated viral outbreaks linked to cruise ships during 2025–2026 (Figure 1), highlighting the increasing public health burden posed by both highly transmissible enteric viruses and emerging zoonotic pathogens in confined travel environments. Norovirus remained the predominant pathogen associated with cruise ship outbreaks, with multiple incidents reported across international cruise liners including Rotterdam, Celebrity Eclipse, AIDAdiva, Oceania Insignia, and several Royal Caribbean and Holland America vessels [11]. These outbreaks primarily presented with acute gastroenteritis symptoms such as vomiting, diarrhoea, nausea, and abdominal cramps, affecting substantial numbers of passengers and crew members.

**TABLE 1:** Travel‐related viral outbreaks (2025–2026): Norovirus [11] and Andes hantavirus [12].

Year	Virus	Location/Setting	Predominant symptoms	Cases/Infected	Deaths	Prevention and response plan
2025	Norovirus	Rotterdam cruise ship	Vomiting, diarrhoea	85/2593 (passengers) 9/1005 (crew)	Not reported	Increased cleaning and disinfection procedures
2025	Norovirus	Celebrity eclipse cruise ship	Vomiting, diarrhoea, abdominal cramps	95/3042 (passengers) 9/1235 (crew)	Not reported	Increased cleaning and disinfection procedures
2025	Norovirus	AIDAdiva cruise ship	Diarrhoea, vomiting	106/2007 (passengers) 8/640 (crew)	Not reported	Increased cleaning and disinfection procedures
2025	Norovirus	Oceania insignia	Diarrhoea, vomiting	88/637 (passengers) 8/391 (crew)	Not reported	Increased cleaning and disinfection procedures
2025	Norovirus	Royal caribbean international	Diarrhoea, vomiting	128/1874 (passengers) 7/883 (crew)	Not reported	Increased cleaning and disinfection procedures
2025	Norovirus	Royal caribbean international	Diarrhoea, vomiting, abdominal cramps	134/3914 (passengers) 7/1266 (crew)	Not reported	Increased cleaning and disinfection procedures
2025	Norovirus	Holland America line	Diarrhoea, vomiting, abdominal cramps	148/2038 (passengers) 22/830 (crew)	Not reported	Increased cleaning and disinfection procedures
2025	Norovirus	Viking expedition operations	Vomiting, diarrhoea	37/355 (passengers) 6/260 (crew)	Not reported	Increased cleaning and disinfection procedures
2025	Norovirus	Seabourn cruise line	Vomiting, diarrhoea, abdominal cramps	13/461 (passengers) 6/405 (crew)	Not reported	Increased cleaning and disinfection procedures
2026	Norovirus	Caribbean princess	Diarrhoea, vomiting	145/3116 (passengers) 15/1131 (crew)	Not reported	Increased cleaning and disinfection procedures
2026	Norovirus	Princess cruises	Diarrhoea, vomiting	141/4307 (passengers) 52/1561 (crew)	Not reported	Increased cleaning and disinfection procedures
2026	Andes virus	MV hondius cruise		11 cases	3	Maintain strict hygiene and infection control measures, including hand hygiene, respiratory etiquette, masking when symptomatic, environmental cleaning, and isolation of suspected cases. Monitor exposed individuals for 42 days, conduct contact tracing, quarantine high‐risk contacts, and seek immediate medical care if symptoms develop. Reduce exposure to rodents and contaminated environments, particularly during rural, occupational, or ecotourism activities. Strengthen public health communication, early detection, supportive clinical management, and coordinated international surveillance and response efforts.

**FIGURE 2:**
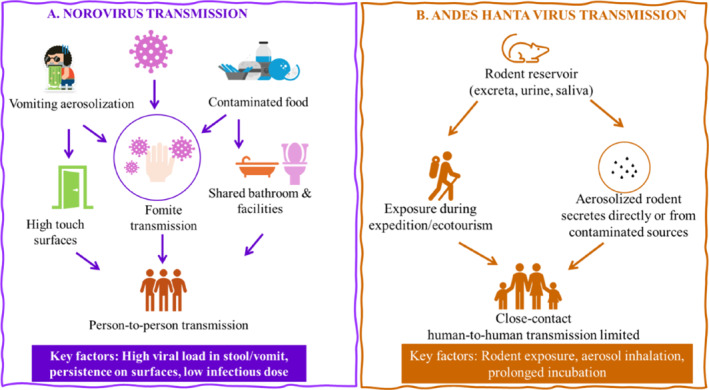
Possible mode of transmission of viruses on cruise ships.

The rapid spread of norovirus aboard cruise ships is strongly associated with its extremely low infectious dose, high shedding rate, and remarkable environmental persistence. Previous studies have demonstrated that norovirus can survive on contaminated surfaces for prolonged periods and remain resistant to several commonly used disinfectants, allowing indirect transmission through fomites such as door handles, elevators, dining areas, and shared restroom facilities [4, 13]. In addition, aerosolisation of viral particles during vomiting episodes may further contribute to widespread environmental contamination, particularly in enclosed settings with limited personal space and centralised ventilation systems [14] (Figure 2).

In contrast, the 2026 Andes hantavirus outbreak associated with the MV Hondius cruise highlighted a different but equally important infectious disease concern linked to expedition‐style travel and ecotourism activities. Although hantaviruses are primarily transmitted through inhalation of aerosolised rodent urine, saliva, or faeces [15], Andes virus is unique among hantaviruses because limited human‐to‐human transmission has been documented, particularly following prolonged close contact with infected individuals [16] (Figure 2). The outbreak raised concerns regarding zoonotic exposure, prolonged incubation periods, delayed symptom onset, and the potential for international dissemination following passenger travel. Multiple confirmed cases and three reported deaths prompted coordinated international public health responses involving contact tracing, quarantine of high‐risk individuals, extended symptom monitoring, and strengthened surveillance across multiple countries. Although such outbreaks remain relatively uncommon, they serve as an important warning of the continuing threat posed by emerging and re‐emerging pathogens capable of causing severe public health consequences.

Collectively, these outbreaks demonstrate how cruise ships and other confined travel environments can amplify viral transmission through high population density, shared facilities, extensive interpersonal interactions, environmental contamination, and international mobility. Consequently, repeated outbreaks have continued to occur despite implementation of enhanced cleaning, disinfection, and infection‐control measures, suggesting that conventional reactive sanitation approaches alone may be insufficient to fully interrupt indirect environmental transmission cycles in maritime settings. These factors further highlight the urgent need for improved preparedness strategies and innovative environmental interventions capable of reducing indirect viral transmission in crowded and highly interconnected settings.

## 3 Available Therapeutics and Current Prevention Strategies

Currently, no specific antiviral therapy, including vaccines, has been fully approved for either norovirus or hantavirus infections, and clinical management primarily relies on supportive care and rigorous infection prevention strategies.

For norovirus infection, treatment mainly includes oral or intravenous rehydration, electrolyte replacement, and symptomatic management, particularly in elderly and immunocompromised individuals who are at higher risk of severe dehydration [17]. Despite extensive research, the development of effective norovirus therapeutics remains challenging because of the virus's high genetic diversity, rapid mutation rate, and lack of robust long‐term culture systems [18]. Several approaches are currently being explored to identify effective therapeutics against norovirus like viral polymerase inhibitors [19], monoclonal antibodies [20], and probiotics [21]. In addition, as of April 2025, at least two norovirus vaccine candidates are undergoing clinical trials [22].

In contrast, management of hantavirus cardiopulmonary syndrome (HCPS) caused by Andes virus primarily depends on intensive supportive care, including oxygen supplementation, haemodynamic monitoring, mechanical ventilation, and extracorporeal membrane oxygenation (ECMO) in severe cases [23]. Although ribavirin has shown partial efficacy against some hantaviruses associated with haemorrhagic fever with renal syndrome, its effectiveness against HCPS remains limited and inconclusive [6].

Given the absence of highly effective antiviral therapies, prevention remains the primary defence against outbreak control in cruise ship settings. According to world health organisation (WHO) and centres for disease control and prevention (CDC) current control measures largely rely on strict hand hygiene, rapid isolation of symptomatic individuals, enhanced environmental cleaning and disinfection, safe food‐handling practices, respiratory etiquette, and onboard surveillance systems. However, the continued recurrence of outbreaks despite repeated implementation of these measures highlights the limitations of conventional sanitation approaches in highly crowded and confined environments, where environmental persistence and indirect transmission can sustain viral spread. Nevertheless, the repeated emergence of viral outbreaks in cruise‐associated settings underscores the urgent need to move beyond solely reactive infection‐control strategies and explore emerging technologies capable of providing continuous passive protection. Advanced environmental interventions, including antiviral surface coatings, self‐disinfecting smart materials, air decontamination systems, may offer promising complementary approaches for reducing indirect viral transmission and strengthening future outbreak preparedness in confined environments.

## 4 Emerging Technologies for Inhibiting Viral Transmission and Enhancing Passive Protection

Despite the application of conventional hygiene and sanitation methods, the continuous incidence of viral outbreaks across cruise ships highlights the urgent need for advanced environmental intervention strategies capable of reducing indirect virus transmission and providing passive protection in closed area. Some examples of recent and advanced air decontamination technologies and antiviral surface modification strategies are summarised in Table 2. These studies have shown promising antiviral efficacy against different viruses.

**TABLE 2:** Examples of some recent air decontamination technologies and smart antiviral surface coatings for viral transmission prevention.

Category	Technology/Material	Proposed mechanism	Virus tested	Key findings/Features
Air decontamination technologies	HEPA filtration [24]	Mechanical filtration of aerosolised particles and virus‐containing droplets	SARS‐CoV‐2	HEPA combined with UV‐C effectively removed airborne SARS‐CoV‐2 bioaerosols in experimental systems
	UV‐C irradiation [25]	UV‐C damages viral nucleic acids and prevents replication	SARS‐CoV‐2, human coronaviruses	Rapid inactivation of airborne and surface‐associated coronaviruses; widely adopted for indoor disinfection
	Photocatalytic oxidation (TiO_2_‐based) [26]	Reactive oxygen species generated under light exposure oxidise viral proteins, RNA, and membranes	SARS‐CoV‐2	TiO_2_ photocatalysis achieved ∼99.9% viral reduction and damaged viral RNA/protein structures
	Plasma sterilisation [27]	Reactive oxygen/nitrogen species damage viral proteins and envelopes	SARS‐CoV‐2	Plasma treatment altered viral morphology and reduced infectivity while maintaining acceptable safety profiles
		Positive and negative ions interact with airborne particles and viral proteins leading to inactivation	HCoV‐229E, SARS‐CoV‐2	Demonstrated antiviral activity and particle reduction; concerns remain regarding by‐product generation and real‐world efficacy
	Photon‐mediated electron emitter (PMEE)/ViRaTon^PBS^ [28]	Low‐energy hyper‐charged photoelectrons generate nano‐electric fields that destabilise viral spike proteins	MHV‐1 (SARS‐CoV‐2 surrogate)	Reduced aerosolised coronavirus infectivity by up to 84% at 1–1.5 m and surface‐associated virus by up to 77% on steel surfaces; operates without ozone or UV‐C production
Antiviral surface coatings & self‐disinfecting smart materials	Metal‐based coatings (copper, zinc, Silver) [29]	Release of metal ions and ROS causing membrane disruption and genome degradation	SARS‐CoV‐2, influenza virus, norovirus surrogates	Copper and silver surfaces showed strong broad‐spectrum antiviral activity with rapid viral inactivation
	Polymer‐based antimicrobial coatings [30]	Hydrophilic anti‐adhesion layers, electrostatic interaction, membrane disruption, and contact killing	SARS‐CoV‐2, influenza virus, HCoV‐OC43, Lentivirus‐EGFP, T4D bacteriophage	Durable coatings effective against bacteria and enveloped viruses; applicable to masks, filters, and medical devices
	Peptide‐coated surfaces [31]	Cationic peptide targeted non enveloped virus capsid while peptide mimics targeted envelope viruses	MHV‐1 (surrogate coronavirus), influenza virus H1N1, murine norovirus type‐1, human adenovirus type 5	Broad‐spectrum antiviral activity with good biocompatibility
	Peptide‐polymer hybrid systems [32]	Peptide‐mediated viral membrane disruption combined with polymer‐based surface stabilisation, sustained release, and anti‐adhesion effects	SARS‐CoV‐2, influenza viruses	Hybrid systems improved antiviral durability, enhanced surface retention, and provided broad‐spectrum antiviral activity suitable for coatings, PPE, and biomedical surfaces
	Nanostructured coatings (graphene‐, silver‐, and metal oxide‐based nanostructured antiviral coatings) [33]	ROS generation, membrane disruption, photothermal effects, and antiviral surface interactions	SARS‐CoV‐2, influenza viruses	Nanostructured coatings improved antiviral efficiency on PPE, filters, and environmental surfaces

Among air decontamination methods, HEPA filter combined with UV‐C irradiation have shown effective removal and inactivation of SARS‐CoV‐2 bioaerosols [24], whereas UV‐C irradiation alone rapidly damages viral nucleic acids and prevents viral replication in both aerosolised and surface‐associated coronaviruses [25]. Another method is photocatalytic oxidation systems utilising TiO_2_ generate reactive oxygen species (ROS) capable of oxidizing viral proteins, RNA, and membranes, achieving up to 99.9% viral reduction against SARS‐CoV‐2 ^26^. Similarly, plasma sterilisation technologies generate reactive oxygen and nitrogen species that disrupt viral proteins and envelopes, significantly reducing SARS‐CoV‐2 infectivity [27]. Bipolar ionization systems have also demonstrated antiviral activity against HCoV‐229E and SARS‐CoV‐2 through ion‐mediated destabilisation of airborne viral particles, although concerns regarding by‐product generation and real‐world efficacy remain [34]. Furthermore, novel photon‐mediated electron emitter (PMEE) systems such as ViRaTon^PBS^ have demonstrated substantial reductions in both aerosolised and surface‐associated coronavirus infectivity by generating hyper‐charged photoelectrons that destabilise viral spike proteins [28]. Importantly, this systems operate without ozone or UV‐C production and may provide continuous passive environmental protection in occupied indoor spaces.

In parallel, essential oil vapour‐based approaches have also demonstrated promising antimicrobial and antiviral activity against airborne pathogens. Vapours containing essential oils significantly reduced culturable aerosolised coronavirus, influenza A virus, bronchitis virus, bacteria, and fungal spores, highlighting their potential as complementary air decontamination strategies in indoor and cruise‐associated settings [35, 36, 37, 38, 39].

Significant advances have also been achieved in antiviral surface coatings and self‐disinfecting smart materials designed to reduce viral persistence on high‐touch surfaces. Metal‐based coatings incorporating copper, zinc, and silver have demonstrated broad‐spectrum antiviral activity against SARS‐CoV‐2, influenza viruses, and norovirus surrogates through metal ion release and ROS‐mediated membrane disruption [29]. Polymer‐based antimicrobial coatings utilise hydrophilic anti‐adhesion layers, electrostatic interactions, and contact‐killing mechanisms to inhibit viral attachment and transmission, with demonstrated activity against SARS‐CoV‐2, influenza viruses, HCoV‐OC43, lentivirus models, and bacteriophages [30]. Peptide‐coated surfaces represent another promising strategy, where cationic peptides and peptide mimics selectively target viral capsids or lipid envelopes, exhibiting broad‐spectrum antiviral activity against surrogate coronaviruses, influenza virus H1N1, murine norovirus, and adenovirus with good biocompatibility [31]. Emerging peptide‐polymer hybrid systems further enhance antiviral durability and surface retention by combining peptide‐mediated viral membrane disruption with polymer‐based stabilisation and sustained‐release properties, making them attractive for coatings, personal protective equipment, and biomedical applications [32]. Additionally, nanostructured coatings incorporating graphene, silver nanoparticles, and metal oxides have shown improved antiviral efficiency through ROS generation, photothermal effects, and direct viral membrane disruption on filters, PPE, and environmental surfaces [33].

**FIGURE 3:**
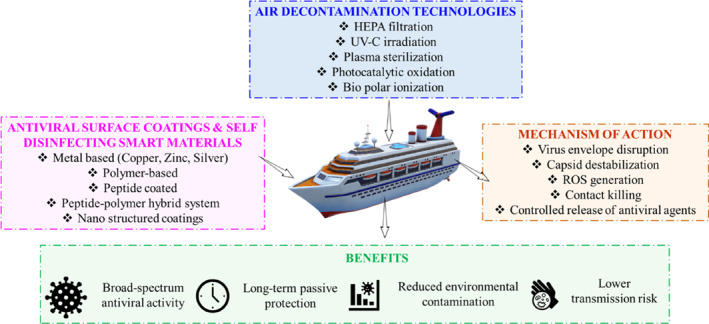
Emerging environmental decontamination and antiviral surface protection strategies.

Collectively, these technologies highlight the growing potential of passive environmental antiviral strategies for reducing viral persistence, environmental contamination, and indirect transmission risk in cruise ships and other densely populated confined environments (Figure 3).

## 5 Future Research Directions

**FIGURE 4:**
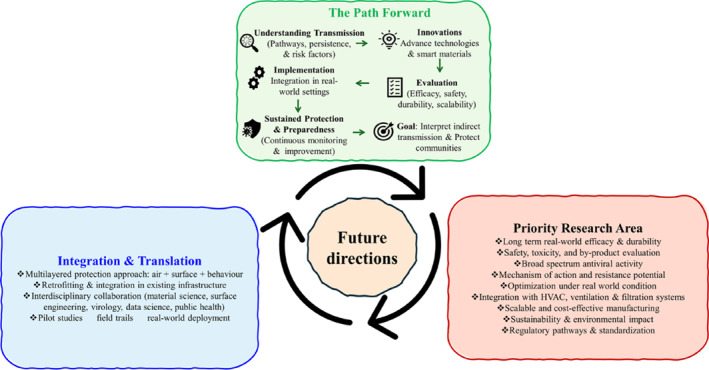
Integrated stratigies for future viral related outbreaks preparedness and prevention.

Virus‐related outbreaks and transmission events will continue to emerge repeatedly, particularly in confined, crowded, and highly interconnected environments. Therefore, it is essential to shift future preparedness strategies beyond conventional outbreak‐response measures and towards integrated technologies capable of reducing indirect viral transmission in cruise ship settings and other closed environments. As presented in Figure 4, understanding viral transmission pathways, environmental persistence, and associated risk factors is fundamental for developing targeted and effective intervention strategies. Emerging innovations such as antiviral surface coatings, self‐disinfecting smart materials, advanced air filtration systems, UV‐based disinfection technologies, and real‐time environmental monitoring platforms may provide additional layers of protection against virus spread in high‐density settings such as cruise ships.

Future research should prioritise the long‐term efficacy, durability, and safety of these technologies under real‐world maritime conditions, including continuous exposure to humidity, salt, cleaning agents, and high passenger turnover. In addition, investigations into broad‐spectrum antiviral activity, mechanisms of action, resistance potential, and potential by‐product toxicity remain essential before large‐scale implementation. Translational research should further emphasise interdisciplinary collaboration across engineering, virology, material science, public health, and environmental science to develop multilayered infection‐control systems integrating air, surface, and behavioural interventions. Pilot studies and field‐based evaluations in active closed settings are also necessary to assess scalability, cost‐effectiveness, sustainability, and regulatory feasibility. Collectively, these approaches may strengthen outbreak preparedness, minimise environmental transmission, and improve public health resilience not only for cruise ship‐associated outbreaks, but also for future viral outbreaks occurring in confined, closed, and crowded environments worldwide.

## 6 Conclusion

The recurrent occurrence of norovirus outbreaks and the emergence of the Andes hantavirus outbreak linked to cruise‐associated travel demonstrate the growing complexity of infectious disease threats in confined and crowed environments. Although the transmission dynamics of these viruses differ considerably, both outbreaks highlight the vulnerability of cruise ships to rapid pathogen dissemination, delayed outbreak detection, and challenges associated with outbreak containment. Current prevention strategies continue to rely heavily on intensified cleaning and disinfection procedures, passenger isolation, and contact tracing. However, the persistence of outbreaks despite repeated implementation of these measures suggests that traditional sanitation practices alone may not be sufficient to effectively interrupt indirect environmental transmission pathways. Increasing evidence supports the important role of contaminated surfaces, aerosolised viral particles, and shared environmental reservoirs in sustaining viral transmission within confined settings. Consequently, emerging environmental intervention technologies, including advanced air decontamination systems, antiviral coatings, self‐disinfecting materials, and peptide‐based antiviral surfaces, represent promising complementary strategies for reducing environmental viral burden and providing continuous passive protection. While many of these technologies remain in early translational stages, they offer important opportunities to strengthen future outbreak preparedness and reduce transmission risk in cruise ships and other crowded environments. Future research should prioritise these approaches to support the development of more resilient and proactive infection‐control frameworks for emerging viral threats.

## Author Contribution

**Umme Laila Urmi:** conceptualisation, investigation, formal analysis, and writing – original draft. **Mark D. P. Willcox:** conceptualisation, supervision, writing – reviewing and editing. All authors have read and agreed to the published version of the manuscript.

## Funding

The authors have nothing to report.

## Conflicts of Interest

The authors declare no conflicts of interest.

## Data Availability

The data that support the findings of this study are available in CDC, ECDPC, WHO at https://www.cdc.gov/norovirus/outbreak‐basics/index.html, reference number 11, 14, 15. These data were derived from the following resources available in the public domain: 1, https://www.cdc.gov/norovirus/outbreak‐basics/index.html; 2, https://www.ecdc.europa.eu/en/infectious‐disease‐topics/hantavirus‐infection/surveillance‐and‐updates/andes‐hantavirus‐outbreak; 3, https://www.who.int/news‐room/fact‐sheets/detail/hantavirus?utm_source=chatgpt.com.
